# Epinephrine-Induced Stemi Mimic Following Robotic-Assisted Bronchoscopy for Pulmonary Nodule Biopsy: A Two-Case Series

**DOI:** 10.7759/cureus.112150

**Published:** 2026-07-06

**Authors:** Joshua Wortsman, Syed M Naqvi, Jay Xiong, Khalid Al Efraij, Sushant Nanavati, Hasnain Bawaadam

**Affiliations:** 1 Pulmonary Medicine, Chicago Medical School, Rosalind Franklin University of Medicine and Science, North Chicago, USA; 2 Pulmonary and Critical Care Medicine, Covenant HealthCare, Saginaw, USA; 3 Interventional Pulmonary and Critical Care Medicine, Advocate Aurora Medical Center, Kenosha, USA

**Keywords:** bronchoscopy, coronary vasospasm, epinephrine, phenylephrine, robotic assisted bronchoscopy, transbronchial biopsy

## Abstract

Topical endobronchial epinephrine is widely used during bronchoscopy for the management of procedure-related bleeding and is generally considered a safe and effective hemostatic agent; however, systemic absorption may rarely result in significant cardiovascular complications. We report two patients who underwent robotic-assisted bronchoscopy with transbronchial biopsy and developed acute ST-segment elevation immediately after endobronchial epinephrine was administered for post-biopsy bleeding. In both cases, electrocardiographic and telemetry findings prompted emergent cardiology evaluation and coronary angiography for suspected ST-segment elevation myocardial infarction. Coronary angiography demonstrated no obstructive coronary artery disease, and the electrocardiographic abnormalities resolved spontaneously without coronary intervention or specific treatment for myocardial ischemia. Both patients recovered without persistent cardiovascular sequelae during the remainder of their hospitalization. The close temporal relationship between epinephrine administration and ischemic electrocardiographic changes, combined with normal coronary angiography, suggests that transient coronary vasospasm was the presumed mechanism, although other causes of transient ST-segment elevation cannot be completely excluded. Although uncommon, this complication has important implications as advanced bronchoscopic procedures increasingly rely on transbronchial sampling techniques that may require topical hemostatic agents. Phenylephrine represents a potentially safer alternative hemostatic agent because it provides local vasoconstriction without β-adrenergic stimulation, which may reduce the risk of arrhythmogenic and ischemic cardiovascular effects. Bronchoscopists should remain vigilant for cardiovascular complications following airway epinephrine administration, particularly in patients with underlying cardiovascular risk factors. Further studies are needed to compare the safety and efficacy of topical epinephrine and phenylephrine in the bronchoscopic setting.

## Introduction

Topical endobronchial epinephrine is widely utilized as a hemostatic agent to manage procedure-related airway bleeding following transbronchial and endobronchial biopsies [1-3]. Epinephrine achieves hemostasis primarily through α₁-mediated vasoconstriction while also possessing β₁-adrenergic chronotropic and inotropic properties [1,2]. Although topical administration is generally considered safe, systemic absorption may rarely result in significant cardiovascular complications, including tachyarrhythmias, coronary vasospasm, and acute myocardial ischemia [1,2,4]. The risk of systemic absorption may be increased when epinephrine is instilled into the distal airways, such as during peripheral biopsies performed using a guide sheath or robotic-assisted bronchoscopy, although clinical data supporting this association remain limited [2,3,5]. Phenylephrine is a selective α₁-adrenergic agonist that also provides local vasoconstriction but lacks β₁-mediated chronotropic and inotropic effects, making it a potential alternative hemostatic agent; however, comparative safety data in bronchoscopy are limited [1,2]. We describe two cases of transient ST-segment elevation myocardial infarction (STEMI) physiology occurring immediately following topical epinephrine administration for hemostasis during robotic-assisted bronchoscopy. These cases highlight a rare but potentially important cardiovascular complication and underscore the need for further investigation into the comparative safety of topical hemostatic agents during advanced bronchoscopic procedures.

## Case presentation

Two patients undergoing robotic-assisted bronchoscopy using the Ion platform for the evaluation of pulmonary nodules developed acute electrocardiographic changes immediately following the administration of topical epinephrine (at a concentration of 0.05 mg/mL) for hemostasis after transbronchial biopsy.

Case one

A 74-year-old man with a 60-pack-year smoking history, chronic obstructive pulmonary disease, an infrarenal abdominal aortic aneurysm, and prior atrial septal defect/patent foramen ovale closure underwent robotic-assisted bronchoscopy using the Ion platform under general anesthesia for biopsy of an enlarging 1.4 cm right lower lobe pulmonary nodule. His baseline electrocardiogram demonstrated normal sinus rhythm with evidence of a remote inferior myocardial infarction, unchanged from prior studies. Following transbronchial biopsy, procedure-related bleeding was treated with 4 mL of topical endobronchial epinephrine (0.05 mg/mL; total dose 0.2 mg). Immediately thereafter, the patient developed hypotension, bradycardia, and marked ST-segment elevation involving the inferior leads (II, III, and aVF) with new atrial fibrillation and rapid ventricular response. Oxygen saturation remained stable throughout the event. Emergent coronary angiography demonstrated no obstructive coronary artery disease, normal right- and left-sided filling pressures, normal pulmonary pressures, and preserved left ventricular systolic function (LVEF 50%) with focal apical hypokinesis. Peak high-sensitivity troponin measured the following day was 522.

**Figure 1:**
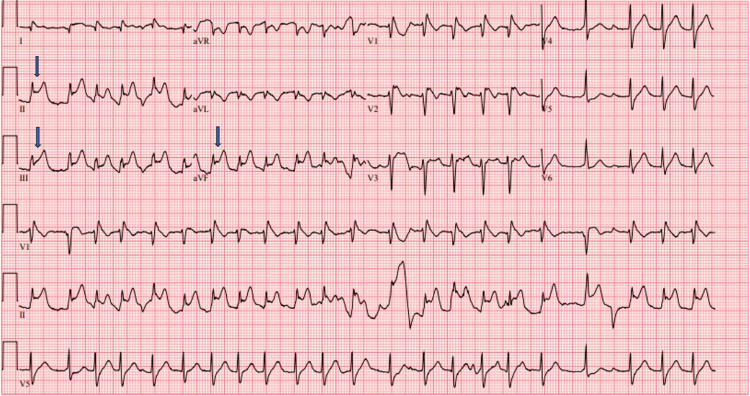
Electrocardiogram obtained immediately following topical endobronchial epinephrine administration during robotic-assisted bronchoscopy in patient 1. Blue arrows highlight acute ST-segment elevation in the inferior leads (II, III, and aVF).

Case two

A 61-year-old woman with a 45-pack-year smoking history and coronary artery disease status post percutaneous coronary intervention with a mid-left anterior descending artery stent underwent robotic-assisted bronchoscopy under general anesthesia for transbronchial biopsy of a positron emission tomography (PET)-avid right middle lobe pulmonary nodule. Her baseline electrocardiogram demonstrated normal sinus rhythm with a possible remote inferior myocardial infarction. Home cardiovascular medications included aspirin, metoprolol, lisinopril, ezetimibe, rosuvastatin, and sublingual nitroglycerin as needed. Following biopsy, procedure-related bleeding was treated with 4 mL of topical endobronchial epinephrine (0.05 mg/mL; total dose 0.2 mg). Shortly thereafter, telemetry and 12-lead electrocardiography demonstrated significant ST-segment elevation with ischemic morphology accompanied by bradycardia, while blood pressure and oxygen saturation remained stable. Emergent coronary angiography demonstrated a patent mid-left anterior descending artery stent, mild non-obstructive coronary artery disease (20-30% proximal right coronary artery stenosis and 50% stenosis of a small-caliber right posterior descending artery), and preserved left ventricular systolic function (LVEF 60-65%). The treating cardiology team considered the transient ST-segment elevation most consistent with coronary vasospasm and recommended treatment with nitrates and a calcium-channel blocker. Troponin was not obtained.

**Figure 2:**
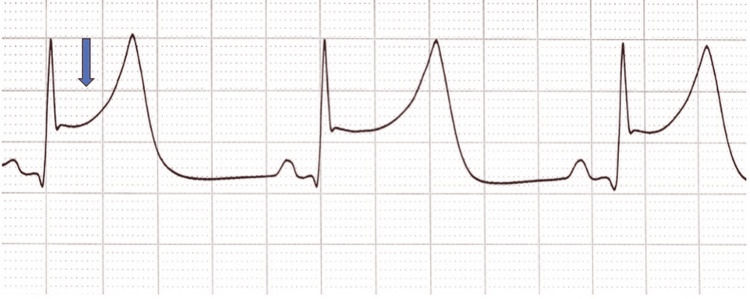
Rhythm strip from patient 2 obtained immediately following topical endobronchial epinephrine administration during bronchoscopy. The blue arrow highlights acute ST-segment elevation.

In both patients, the electrocardiographic abnormalities resolved spontaneously, and coronary angiography demonstrated no obstructive coronary artery disease. These findings are consistent with transient coronary vasospasm or catecholamine-mediated myocardial injury, although neither mechanism was directly confirmed.

## Discussion

The management of iatrogenic airway bleeding following transbronchial or endobronchial biopsy requires a careful balance between effective hemostasis and cardiovascular safety. Although bronchoscopy itself has rarely been associated with transient ST-segment elevation, coronary vasospasm, acute left ventricular dysfunction, and systemic air embolism, the close temporal relationship between topical endobronchial epinephrine administration and the immediate onset of electrocardiographic abnormalities in both of our patients suggests that epinephrine was the most likely precipitating factor. This interpretation is further supported by the absence of obstructive coronary artery disease on emergent coronary angiography and is consistent with previously reported cases of topical endobronchial epinephrine-associated cardiovascular complications [4-7]. Nevertheless, given the observational nature of this two-patient case series, alternative mechanisms cannot be completely excluded.

The literature highlights that topical endobronchial epinephrine can rarely precipitate serious cardiovascular complications, including ventricular arrhythmias, coronary vasospasm, and transient ST-segment elevation mimicking acute myocardial infarction [1,4,5]. Previous reports have described significant ST-segment elevation and coronary vasospasm following administration of 3 mL of 1:10,000 epinephrine (0.3 mg), while myocardial infarction has been reported after a peripheral submucosal dose as low as 0.036 mg [4,5]. In our cases, both patients received 4 mL of 1:20,000 epinephrine (total dose 0.2 mg) immediately before the onset of electrocardiographic abnormalities. Although this dose is lower than that reported in some cases of coronary vasospasm, it exceeds the lowest reported dose associated with myocardial injury, further illustrating that clinically significant cardiovascular events may occur even with relatively small amounts of topical epinephrine. Systemic absorption may be increased when epinephrine is administered into the distal airways because of the rich pulmonary capillary network, particularly in the setting of active mucosal bleeding, although the extent of absorption is likely variable and difficult to predict [3,5].

The role of topical epinephrine in bronchoscopy continues to evolve. Earlier studies in selected patient populations suggested that prophylactic administration of highly diluted epinephrine before transbronchial biopsy may reduce the incidence of severe hemorrhage. However, a recent multicenter randomized trial demonstrated that prophylactic adrenaline instillation did not significantly reduce clinically important biopsy-related bleeding in a broader patient population [3,8]. Importantly, these findings relate specifically to prophylactic administration and should not be directly extrapolated to the reactive treatment of established airway bleeding, where evidence remains limited. Although topical epinephrine continues to be used as rescue therapy in selected cases, clinicians should carefully weigh its potential hemostatic benefit against the possibility of systemic cardiovascular adverse effects, particularly in patients with underlying cardiovascular disease or other risk factors for arrhythmia [9].

Management of procedure-related airway bleeding during bronchoscopy involves a stepwise approach that may include bronchoscope wedging, cold saline instillation, topical vasoconstrictors, tranexamic acid, balloon occlusion, and other adjunctive hemostatic techniques, depending on the severity and location of bleeding. Among topical vasoconstrictors, phenylephrine represents a potential alternative to epinephrine. As a selective α₁-adrenergic agonist, phenylephrine provides local vasoconstriction without β₁-mediated chronotropic or inotropic effects, thereby avoiding the direct cardiostimulatory properties of epinephrine [1,2]. Physiologic studies have demonstrated that inhaled phenylephrine increases local bronchovascular resistance without significant changes in systemic vascular resistance or blood pressure [10]. In addition, retrospective studies have reported a favorable hemodynamic profile, with only modest transient increases in mean arterial pressure and no clinically significant increase in cardiovascular complications compared with control groups [1,2]. Although these findings are encouraging, prospective comparative studies evaluating topical phenylephrine versus epinephrine in bronchoscopy are lacking.

Although caution should be exercised in patients with severe baseline hypertension because phenylephrine may transiently increase mean arterial pressure [2,9], and rare but serious adverse events, including hypertensive crisis and pulmonary edema, have been reported in the otolaryngology literature [11], its lack of β-adrenergic stimulation makes it an attractive topical vasoconstrictor for airway bleeding. At our institution, these pharmacologic considerations, together with the cases presented here, have led to the adoption of topical phenylephrine (0.1 mg/mL) as an alternative hemostatic agent in selected patients. However, larger prospective studies comparing topical phenylephrine with epinephrine are needed to better define their relative safety and efficacy and to guide evidence-based recommendations for the management of procedure-related airway bleeding during bronchoscopy.

This report has several limitations. It describes only two patients and therefore cannot establish the incidence of this complication or a causal relationship between topical endobronchial epinephrine administration and transient ST-segment elevation. Systemic epinephrine concentrations were not measured, precluding confirmation of systemic absorption as the underlying mechanism, and coronary vasospasm was inferred from the clinical presentation and angiographic findings rather than directly demonstrated with provocative testing. Additionally, not all diagnostic data were available for both patients, limiting direct comparison between the cases. Despite these limitations, the close temporal relationship between topical epinephrine administration and electrocardiographic changes in two independent patients, together with the absence of obstructive coronary artery disease on emergent coronary angiography, highlights a rare but clinically important complication that warrants awareness among bronchoscopists.

## Conclusions

These cases highlight a rare but potentially serious cardiovascular complication associated with topical endobronchial epinephrine administration during robotic-assisted bronchoscopy. Although the temporal relationship and angiographic findings suggest transient coronary vasospasm or catecholamine-mediated myocardial injury as the most likely mechanism, causality cannot be definitively established. Bronchoscopists should remain vigilant for cardiovascular complications following topical epinephrine administration, particularly in patients with underlying cardiovascular risk factors. Further prospective studies are needed to compare the safety and efficacy of topical hemostatic agents, including epinephrine and phenylephrine, and to guide evidence-based management of procedure-related airway bleeding.
